# Work of the past Quarter

**Published:** 1918-03-09

**Authors:** 


### THE LONDON HOSPITAL.

### Work of the Past Quarter.

At the Quarterly Court, held on Wednesday, March 6, it was reported that the Quinquennial Appeal was already meeting with substantial response. His Majesty the King has sent a cheque for £500, accompanied by a letter of warm approbation for the work which is being done by the hospital. The Lord Mayor has also given valued assistance by writing a letter to the press advocating the hospital's claims to the generosity of the citizens of London, and as one of the results of his help, Sir Marcus Samuel has offered £1,000 if nineteen other people will do the same. £12,000 has already been promised, including £1,000 from Sir Marcus's brother. The whole appeal has yielded £39,000 this year.

### Romance of a Parcel.

It was announced that the name has been discovered of the donor who some years ago anonymously presented £10,000's worth of Pennsylvania Bonds to the hospital. A parcel which the Secretary took to be of no particular value or interest was put on his table one morning. He was very busy and did not open this parcel for many days, when he was surprised and overjoyed to discover £10,000's worth of bonds wrapped in this very ordinary-looking parcel. The name of the donor must still remain a secret, and it is known only to Lord Knutsford and the Secretary.

The new home for nurses, which by Queen Alexandra's command is named the Cavell Home in memory of Nurse Edith Cavell, will probably be ready for occupation in May next. It has been built in circumstances of the greatest difficulty, but the Government have done all they can to assist by authorising the Ministry of Munitions to give special permits. Although this building has taken a very long time to finish, but for these permits it would have taken very much longer. Sir George Frampton, the eminent sculptor, has most generously presented a bust of the late Miss Cavell, which will be placed in the sitting-room belonging to the nurses.

The King's Fund grant was £15,500, the largest grant they have ever made to the hospital; the amount includes £1,500 for the Cavell Home.

### Treatment of Discharged Sailors and Soldiers.

An agreement is being entered into between the Ministry of Pensions and the hospital, whereby the treatment is undertaken of discharged sailors and soldiers disabled in the service of the nation. The Ministry have agreed to pay the rate of 7s. a day for in-patients and 2s. per attendance for out-patients.

There was in 1917 a substantial falling-off in the number of out-patients treated—viz. : 39,860, as against 67,165 in 1916. The patients passing through the receiving-room remained substantially the same. In-patients were 17,637 in number, comparing with 16,053 in 1916.

### Appointments and Resignations.

Captain F. F. Muecke was appointed officer-in-charge of the officers' wards, but to the regret of the committee, although greatly to his own advantage and advancement, he has been appointed to command all the Flying Corps hospitals for officers in London. Mr. Morris, the House Governor, has been placed on the Advisory Committee for assisting the Ministry of Food in connection with the rationing of hospitals. Miss Gwendolen Powell, the senior lady almoner, has resigned as she is about to be married. Her sister, Miss Norah Powell, has been appointed in charge of the almoner's department. Mr. Sherren, one of the senior surgeons, has been appointed officer-in-charge, with the rank of colonel, of all the officers' beds in London.

Mr. Hills, who has been steward for twenty-five years, has been seriously indisposed, and in consequence the House Committee has granted him leave of absence for one year. The House Committee has appointed Miss Beatrice Monk, formerly chief matron's assistant, to take his place during his enforced absence.

### L.C.C. Cases.

The London County Council has asked for a renewal of the agreement with them to treat certain cases as from April 1 prox. The aural cases will be reduced from 2,500 to 2,000. The L.C.C. guarantee the payment of 200 ringworm cases at 7s. each.

### Blood Transfusion.

The hospital is at present making a register of the names of men who are willing to give blood for the treatment of patients requiring transfusion.

